# *Feminizer* and *doublesex* knock-outs cause honey bees to switch sexes

**DOI:** 10.1371/journal.pbio.3000256

**Published:** 2019-05-06

**Authors:** Alison McAfee, Jeffery S. Pettis, David R. Tarpy, Leonard J. Foster

**Affiliations:** 1 Department of Entomology and Plant Pathology, North Carolina State University, Raleigh, North Carolina, United States of America; 2 Department of Biochemistry and Molecular Biology, University of British Columbia, Vancouver, British Columbia, Canada; 3 Pettis and Associates, Salisbury, Maryland, United States of America

## Abstract

Honey bees are experts at refuting societal norms. Their matriarchal hives are headed by queens, backed by an all-female workforce, and males die soon after copulation. But the biochemical basis of how these distinct castes and sexes (queens, workers, and drones) arise is poorly understood, partly due to a lack of efficient tools for genetic manipulation. Now, Roth and colleagues have used clustered regularly interspaced short palindromic repeats (CRISPR) to knock out two key genes (*feminizer* and *doublesex*) that guide sexual development. Their technique yielded remarkably low rates of genetic mosaicism and offers a promising tool for engineering and phenotyping bees for diverse applications.

## Abstract 2

This Primer explores the implications of a new study that uses CRISPR gene editing to investigate genetic switches controlling sex determination in the honey bee, Apis mellifera.

What has a greater influence on determining someone’s height? Is it how tall their parents are, or the quality of nutrition they receive during key growth periods? For centuries, researchers have been debating the relative importance of nature and nurture—genes and environment—for shaping who we are, from dictating our height to our sexual orientation. But the nature versus nurture argument isn’t only relevant for humans; honey bees, too, are at the intersection of these 2 forces. For them, it dictates their developmental destiny.

## The sex genes

**Fig 1:**
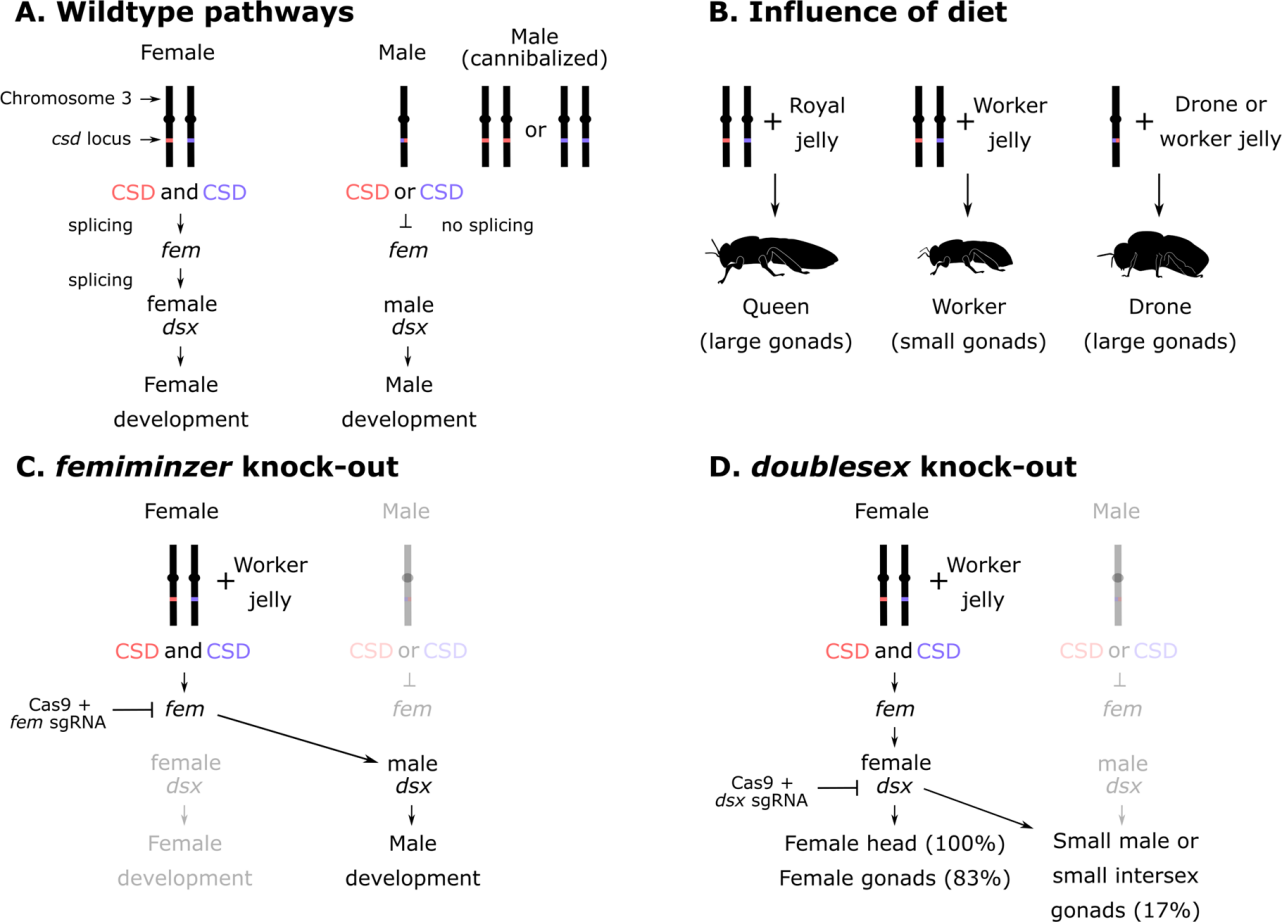
Sex and caste determination in honey bees. (A) Sex determination cascade. (B) General roles of diet in caste determination. Roth and colleagues demonstrated that drones develop normal, large gonads when reared on a worker diet. (C) The effect of *fem *and (D) *dsx* knock-out on sex determination and gonad size, as described by Roth and colleagues (2019). CRISPR, clustered regularly interspaced short palindromic repeats; CSD, complimentary sex determiner; *dsx*, doublesex; *fem*, feminizer; sgRNA, single guide RNA.

Honey bee colonies are comprised of drones (reproductive males), workers (sterile females), and a queen (reproductive female). As early as 1845, Johan Dzierzon—a Polish theologist and one of the founders of modern beekeeping—noticed that virgin queens could only lay eggs that became drones, and from this he deduced that male bees must be haploid [1]. This early inference turned out to be basically true. A bee’s sex is actually determined by the allele(s) at a specific spot in the genome: the complimentary sex determiner locus (the *csd* gene; Fig 1) [2]. This fits perfectly with Dzierzon’s observations—hemizygous (unfertilized) eggs develop as males and heterozygous (fertilized) eggs developing as females. Homozygous eggs also develop as males, but these are rare because the diversity of *csd* alleles is high (there are hundreds of distinct alleles [3]), and workers discriminate against the homozygous larvae by eating them before they can develop [4]. A bee’s sex is thus a consequence of its genotype, falling squarely in the realm of “nature.”

For reasons that are still not entirely clear, the heterozygous *csd* genotype leads to expression of functional CSD proteins—likely acting as heterodimers, although other potential explanations have been proposed [5]—which splice transcripts of a downstream gene, *feminizer* (or *fem*). The spliced Fem protein in turn regulates the splicing of *doublesex* (*dsx*)—another downstream gene—whereas the unspliced Fem is not functional and leaves *dsx* unspliced as well. This cascade is key for proper female and male development [6]. But queens and workers—the female castes—are both diploid (with few exceptions [7,8]) and heterozygous at the *csd* locus. A worker and queen can theoretically be genetically identical. So, what makes one develop into a long-lived, regal queen, with her rounder face and large abdomen housing voluptuous ovaries, instead of a sterile worker? Logically, their genetic similarity should leave just one option: nurture.

## You are what you eat

Nurture, in this case, is an especially apt description. Worker- and queen-destined larvae are fed different diets, and the diet itself acts as a rheostat that dials the royal developmental program up or down. Indeed, dietary components that drive worker and queen differentiation have been identified [9,10], but their omnipotent role is hotly debated [11]. Queens receive excess amounts of royal jelly (a blend of secretions from the workers’ hypopharangeal gland, postcerebral gland, and thoracic gland), whereas workers receive limited amounts of worker jelly (a blend of pollen, honey, and glandular secretions). An intermediate diet yields intercaste females [12], suggesting that diet-mediated caste determination acts on a continuous scale rather than via binary commitments.

There is no doubt that diet is a driver of caste determination. Different diets cause massive epigenetic changes to the DNA of worker- and queen-destined larvae [13,14] and regulate key developmental genes [9,10]. Other researchers have suggested that the highly nutritious queen food also allows her to develop faster and become larger than her worker counterparts [15]. But the nutritional explanation for worker and queen differentiation is not all there is to it. New evidence, published by Roth and colleagues in this issue of *PLOS Biology*, suggests that the underlying mechanism is even more complex than we thought [16].

Annika Roth, Dr. Martin Beye, and their colleagues at the Heinrich-Heine University, Germany, have elegantly shown that this particular nature versus nurture debate is far from settled [16]. We know that queens and drones both have sugar-rich diets, and both have large gonads, whereas workers have a more limited diet and have underdeveloped gonads. To test whether the worker diet alone was sufficient to give drones little gonads, Roth and colleagues fed wild-type drones a worker diet and recorded the size of their testes. They saw no change in size, suggesting that the diet alone does not control the growth of a drone’s sex organs. Rather, the researchers suspected that diet might intersect with the sex determination pathway, which they investigated using genetic engineering. They employed a technique called CRISPR to achieve this, which uses a bacterial protein (Cas9; acting like DNA-cutting molecular scissors) and a programmable RNA sequence to guide the Cas9 scissors to cut and disrupt the chosen genes (in this case, *fem* and *dsx*). When cells try to repair their broken DNA, they eventually introduce mistakes that render the gene non-functional.

## Double knock-outs

In a sweltering 33°C room, Roth and colleagues painstakingly injected thousands of delicate, freshly laid, genetically female eggs with the Cas9 machinery and guide RNA. The guide RNA targeted the *fem* gene, which is normally functional in genetic females and nonfunctional in genetic males. As they report in the manuscript, anywhere between 60% and 99% of eggs do not survive this process, mainly due to the trauma of being pierced with a glass needle. But enough of them survived for the researchers to phenotype. Despite being genetically female, Roth and colleagues found that when they disrupted *fem*, the genetically female embryos completely switched to males and even grew large male sex organs. This was despite being reared on a nutrient-restricted worker diet. *Fem* is therefore a master regulator and is necessary for restricting the development of sex organs. Because females expressing *fem* have small ovaries on a worker diet and large ovaries on a queen diet, the developmental program induced by *fem* must interact with diet somewhere along the way.

In females, the Fem protein splices *dsx*, but in males, Fem is nonfunctional and *dsx* remains unspliced. When the researchers knocked out *dsx* and reared the larvae on a worker diet, they observed no difference in gonad size between mutated and wild-type workers, despite some of the mutants (17% or 11 bees) being intersexual. In fact, these mutant honey bees had feminine heads but male reproductive organs. Other mutants had female reproductive organs but with missing or deformed ovarioles. But every one of the double-mutants—even those with testes—had small gonads, similar to wild-type workers with functional *dsx*. This observation is again consistent with Fem being necessary for small gonad size, regardless of gonad type.

Without a reliable method of rearing queens in the lab, we still don’t know the role these genes play in queen development or how they interact with queen nutrition. And we still don’t know what other genes and regulatory mechanisms are involved in executing the sex-switching phenotypes that arise from *fem* and *dsx* mutation. But this research offers a classic knock-out investigation of the genetic basis of sex determination—a feat which has rarely been achieved in honey bees. And the progress Roth and colleagues made in engineering efficiency might actually be their biggest impact.

## From transgenes to CRISPR

Dr. Martin Beye’s laboratory specializes in studying sex and caste determination—both of which are topics rooted in basic biology. In fact, Beye wrote the seminal research article describing the *csd* gene back in 2003 [2]. But his research group is also the world leader in honey bee genetic engineering, which they performed for the first time in 2014 [17]. Back then, they used the piggyBac transposon technique to create transgenic bees. However, the CRISPR method is more versatile, and although it has been performed in honey bees before [18,19], Roth’s and Beye’s work has greatly improved the efficiency of mutagenesis.

The piggyBac engineering system demonstrated the proof of principle—that honey bee eggs could be injected with gene-editing molecular machinery, yielding larvae and eventually adult bees with edited genomes [17]. However, all of the piggyBac engineered honey bees were mosaics; that is, some of their cells had the transgene whereas others did not. This meant that most biological experiments were impractical. The mosaic larvae must be reared into queens, and because it cannot be reliably done in the lab, a colony must be coaxed to accept the engineered larva and give her a queen diet. Then the queen’s eggs must be screened for germ-line incorporation. If the incorporation rate was high enough, her transgene-containing drone offspring could be collected along with their germplasm. And this germplasm could then be used for instrumental insemination. Despite interest, this approach has not yet been executed, owing to its complexity, limited duration of field seasons, and high likelihood of failure during at least one step of the process.

With the CRISPR method, however, Roth and colleagues were able to produce complete double-mutant larvae nearly 80% of the time. That is, with a single injection of carefully designed guide RNA and a balanced ratio of Cas9 to guide RNA, both DNA strands in every single nucleus of the developing embryo were mutated at the target site. This contrasts with previous CRISPR-based gene editing in honey bees, which achieved only 5% to 12% germline editing efficiency [18,19]. The impact of these efficiency improvements is hard to understate: if other researchers can achieve this success rate, this will let scientists avoid the logistical nightmare of rearing engineered queens, screening their sons, harvesting the germplasm, instrumental insemination, and establishing the queens in an environmentally isolated colony. Instead, genetic screening and phenotyping can be done directly on the injected larvae, as Roth and colleagues conducted here. The potential applications of this method are endless, from assigning functions to poorly understood genes, to deciphering immune mechanisms, to understanding pathways involved in pesticide toxicity.

The development of CRISPR technology has clearly brought us into a whole new era of functional genomics research. We are no longer bound by the constraints of studying model species, like fruit flies or nematodes, in which genetic tools are relatively simple to execute. Beyond its fascinating biology, the honey bee is also an economically valuable species so there will no doubt soon be efforts to engineer honey bees with industrially beneficial traits, such as pesticide resistance. Such a trait would of course be beneficial to honey bees—and potentially beekeepers—but not the native pollinators that would still be susceptible. Still other traits, like disease resistance (if a simple enough genetic mechanism can be deciphered), may have less ecological risk and a great reward. In every case, we must ask ourselves—as researchers and ambassadors—if the benefit of such modifications outweigh the associated risks. Regardless of industrial applications, the substantial advance in the application of CRISPR in bees reported by Roth and colleagues will undoubtedly lead to many more discoveries about the intricate control of development and social behavior in this fascinating insect.

## Abbreviations

CRISPR: clustered regularly interspaced short palindromic repeats
dsx: doublesex
fem: feminizer
csd: complimentary sex determiner
